# *Clostridium sporogenes* increases fat accumulation in mice by enhancing energy absorption and adipogenesis

**DOI:** 10.1128/spectrum.04116-23

**Published:** 2024-06-25

**Authors:** Lei Du, Jing Wang, Xiaoyu Qiu, Qi Wang, Han Peng, Jinxiu Huang, Feiyun Yang, Zuohua Liu, Renli Qi

**Affiliations:** 1 Chongqing Academy of Animal Science, Chongqing, China; 2 Zhejiang Engineering Research Center for Tissue Repair Materials, Wenzhou Institute, University of Chinese Academy of Sciences, Wenzhou, Zhejiang, China; 3 Sichuan Animal Science Academy, Chengdu, China; 4 National Pig Technology Innovation Center, Chongqing, China; Institute of Microbiology, Chinese Academy of Sciences, Beijing, China

**Keywords:** *Clostridium sporogenes*, gavage experiment, fat accumulation, metagenomics, fecal metabolites

## Abstract

Gut bacteria belonging to the *Clostridium* family play a pivotal role in regulating host energy balance and metabolic homeostasis. As a commensal bacterium, *Clostridium sporogenes* has been implicated in modulating host energy homeostasis, albeit the underlying mechanism remains elusive. Therefore, this study aimed to investigate the impact of *C. sporogenes* supplementation on various physiological parameters, intestinal morphology, particularly adipose tissue accumulation, and glucolipid metabolism in mice. The findings reveal that mice supplemented with *C. sporogenes* for 6 weeks exhibited a notable increase in body weight, fat mass, adipocyte size, and serum triglyceride (TG) levels. Notably, the increased fat accumulation is observed despite consistent feed intake in treated mice. Mechanistically, *C. sporogenes* supplementation significantly improved the structure integrity of intestinal villi and enhanced energy absorption efficiency while reducing excretion of carbohydrates and fatty acids in feces. This was accompanied by upregulation of glucose and fatty acid transporter expression. Furthermore, supplementation with *C. sporogenes* promoted adipogenesis in both liver and adipose tissues, as evidenced by increased levels of hepatic pyruvate, acetyl-CoA, and TG, along with elevated expression levels of genes associated with lipid synthesis. Regarding the microbiological aspect, *C. sporogenes* supplementation correlated with an increased abundance of *Clostridium* genus bacteria and enhanced carbohydrate enzyme activity. In summary, *C. sporogenes* supplementation significantly promotes fat accumulation in mice by augmenting energy absorption and adipogenesis, possibly mediated by the expansion of *Clostridium* bacteria population with robust glycolipid metabolic ability.

**IMPORTANCE:**

The *Clostridia* clusters have been implicated in energy metabolism, the specific species and underlying mechanisms remain unclear. This present study is the first to report *Clostridium sporogenes* is able to affect fat accumulation and glycolipid metabolism. We indicated that gavage of *C. sporogenes* promoted the adipogenesis and fat accumulation in mice by not only increasing the abundance of *Clostridium* bacteria but by also enhancing the metabolic absorption of carbohydrates and fatty acids significantly. Obviously, changes of gut microbiota caused by the *C. sporogenes*, especially the significant increase of *Clostridium* bacteria, contributed to the fat accumulation of mice. In addition, the enhancement of *Clostridium* genus bacteria remarkably improved the synthesis of hepatic pyruvate, acetyl-CoA, and triglyceride levels, as well as reduced the excretion of fecal carbohydrates, short-chain fatty acids, and free fatty acids remarkably. These findings will help us to understand the relationship of specific bacteria and host energy homeostasis.

## INTRODUCTION

Numerous studies have elucidated the intricate interplay between intestinal microbiota and host energy metabolism. Notably, mice raised in a normal environment exhibited 42% and 47% greater combined fat and epididymal fat weight compared to those raised in a sterile environment, with control mice consuming less energy than germ-free mice (1). This suggests that the presence of microbiota significantly enhances diet-induced energy yield. Furthermore, fecal microbiome transplantation from lean donors yielded different outcomes compared to transplantation from obese mice, with the latter showing higher body fat and an elevated Firmicutes to Bacteroidetes (F/B) ratio, indicating a potential role of Firmicutes in augmenting energy extraction from the diet (2, 3). Among Firmicutes, *Clostridium* species stand out for their ability to ferment various nutrients, including carbohydrates, proteins, and organic acids, to produce short-chain fatty acids (SCFAs), thereby enhancing host energy absorption (4, 5). Round et al. demonstrated that germ-free mice colonized with the *Clostridia* cluster can downregulate genes related to lipid uptake and reduce obesity (6). Collectively, these studies indicate that *Clostridium* bacteria play a crucial role in regulating energy metabolism and bodily health through functional metabolites.

This study aims to investigate the impact of *Clostridium sporogenes* on energy metabolism in mice through gavage experiments. *C. sporogenes* was chosen mainly based on previous research indicating its ability to alleviate muscle inflammation and promote lipogenesis in treated mice (7). Several additional factors were also considered in the selection of *C. sporogenes* for study. Firstly, *C. sporogenes* is known for its capacity to synthesize a plethora of small molecules involved in energy metabolism, including ATP and SCFAs, branched chain fatty acids, and aromatic fatty acids through amino acid metabolism *in vitro* (8). Secondly, *C. sporogenes* acts as a non-pathogenic intestinal symbiotic bacterium that is amicable to both humans and animals, without inducing significant immune responses in the intestine. Thirdly, *C. sporogenes* has been demonstrated to efficiently colonize in the intestinal tract of germ-free mice and plays a role in regulating host metabolism (9). Lastly, as a spore-forming species of the *Clostridium* genus, *C. sporogenes* exhibits robust stress resistance, facilitating its establishment and proliferation within the intestinal environment (4).

Our study aims to elucidate the influence of *C. sporogenes* on fat accumulation in mice, shedding light on its role in regulating energy homeostasis. These findings provide novel insights into potential microbial targeted therapies for metabolic disorders.

## MATERIALS AND METHODS

### Mouse treatment and sampling

**Fig 1:**
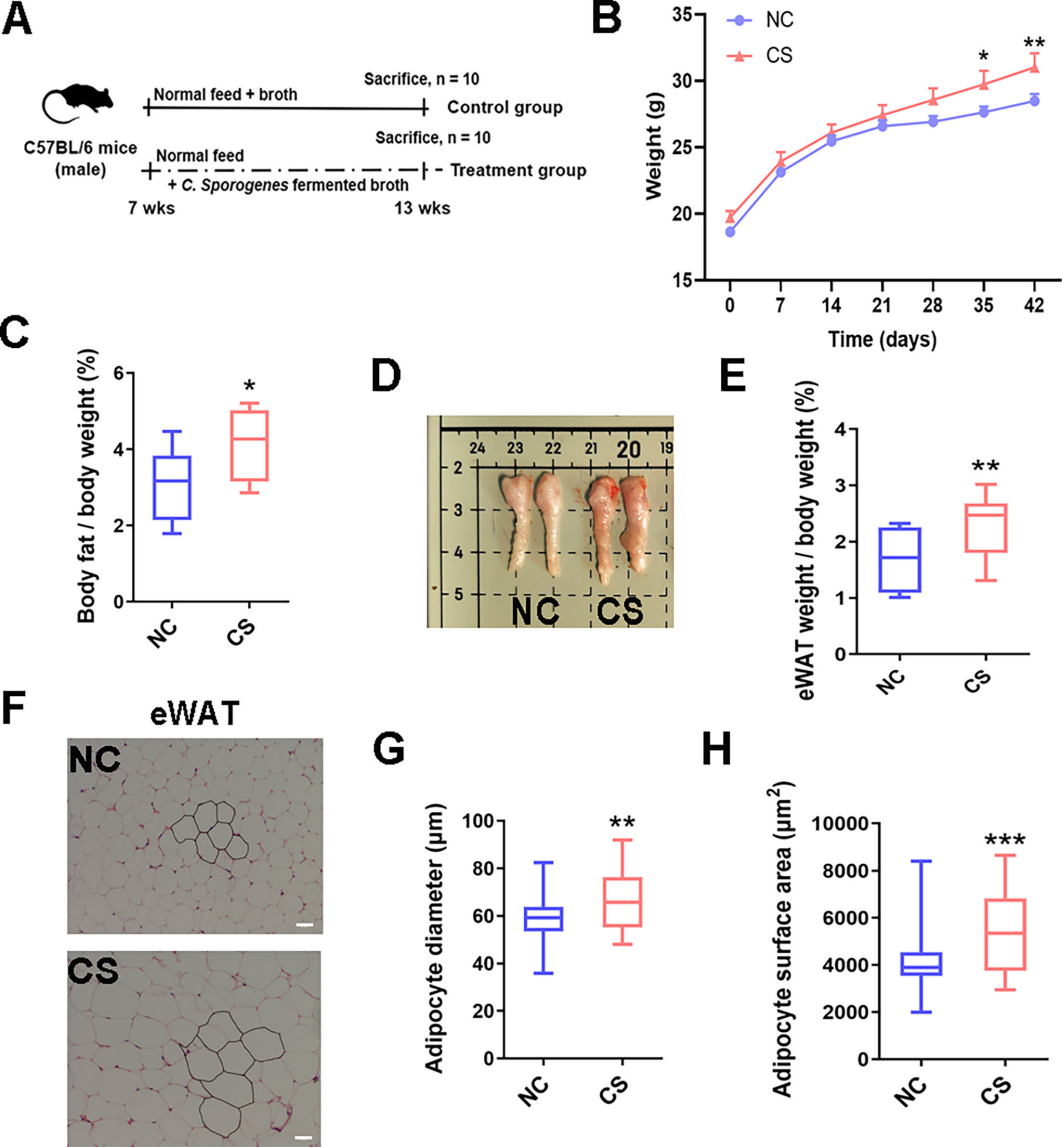
*C. sporogenes* treatment increases lipid deposition and body fat accumulation in the mice after 42 days of oral treatment. (**A**) Grouping and treatment of the mice (*n* = 10). (**B**) Mice weekly body weight gain (*n* = 10). (**C**) Ratio of body fat gain to the weight gain of mice (*n* = 10). (**D**) Size of epididymal white adipose tissue (eWAT) and (**E**) the ratio of eWAT weight gain to the body weight gain of mice (*n* = 10). (**F**) Representative hematoxylin-eosin staining picture of adipose tissue (×200), (**G**) mean adipocyte diameter, (**H**) and adipocyte area of the eWAT in mice (*n* = 6); scale bar, 50 µm. Data are presented as the mean ± SEM, *P < 0.05, **P < 0.01, ***P < 0.001. (*n* = 6–10).

Male C57BL/6 mice aged 6 weeks (Huafukang, Beijing, China) were maintained in groups of up to four mice per cage under controlled conditions with *ad libitum* access to food and water (*n* = 10). Mice were acclimated to the environment for 1 week before the start of the experiment. Subsequently, they were randomly divided into two groups (Fig. 1A), (i) a normal chow diet (D12450J, Paddy Biological Technology Co., Ltd., Chengdu, China) (NC group) and (ii) a normal chow diet with *C. sporogenes* [American Type Culture Collection (ATCC) 15579] via oral gavage (CS group). The NC group received sterile bacteria culture media as a vehicle, whereas mice of the CS group received oral gavage of *C. sporogenes* twice weekly at a dose of 1 × 10^8^ clone formation units (CFUs)/200 µL. Body weight was measured weekly throughout the 6-week trial period.

At 13 weeks of age, all mice were sacrificed, and subcutaneous fat, epididymal white adipose tissue (eWAT), and subscapular brown adipose tissue (sBAT) were collected and weighed. Fat mass gain (%) was calculated as the sum of subcutaneous fat (g), eWAT (g), and sBAT (g) weights divided by body weight. Subsequent analyses were conducted on samples from six mice in each experimental group.

### Detection of serum lipid levels

Serum lipid levels, including triglyceride (TG), total cholesterol (TC), low-density lipoprotein cholesterol (LDL-C) and high-density lipoprotein cholesterol (HDL-C), were measured using an automatic biochemical analyzer (AU5400, Beckman Coulter, CA, China).

### Short-chain fatty acids detection

Fresh fecal samples were mixed with the extract solution (chloroform: acetone = 1:1) at a ratio of 1:1.5 and then incubated at 40°C for 1 h in water bath. The extract solution was carefully absorbed and then centrifuged at 10,000 r/min for 15 min. Gas chromatography (Trace1310, Thermo Scientific, MA, USA) was used to determine the content of SCFAs in the extract according to the internal standard method.

### Detection of liver glycolytic products

Concentrations of glycolytic products, including TG, 6-fructophosphokinase (PFK), pyruvate, and acetyl-CoA, in liver samples were quantified using commercial mouse enzyme-linked immune sorbent assay kits (mlbio Co. Ltd., Shanghai, China).

### Determination of energy efficiency

Fecal energy content was assessed using an isoperibol oxygen bomb calorimeter assembly (A1435DDEE, Parr Instruments Co., Illinois, USA) on fecal samples harvested over 24 h during the 4th week of the experiment. All fecal samples from the mice were collected daily for 7 days and then dried overnight at 60°C in a constant temperature blast oven immediately (DHG-9240A, Shanghai Jinghong Experimental Equipment Co., Ltd., Shanghai, China). Subsequently, the dried samples were ground into a yellowish-green powder using a high-speed crusher (FW80, Tianjin Tester Instrument Co., Ltd., Tianjin, China). The gross energy concentrations of both the feed and fecal dry matter (DM) were determined. Finally, the energy efficiency on a DM basis of mice was calculated using the formula: Energy efficiency (g/kcal) = Body wt gain (g/d) / Mice digestible energy (kcal/d) = Body wt gain (g/d) / [(feed intake × feed energy) − (fecal output × fecal energy)].

### Bacterial strains’ culture

*C. sporogenes* was obtained from the ATCC and cultured with fluid thioglycollate medium and reinforced clostridial agar. The bacterial strain was typically incubated under anaerobic conditions at 37°C for 24–48 h.

### Hematoxylin and eosin staining

The eWAT, sBAT, liver, and jejunum tissues of mice were fixed in 10% neutral formaldehyde at room temperature for 7 days. Following dehydration in graded ethanol, the tissues underwent diaphanization with xylene and then were embedded in paraffin. Subsequently, the muscle and intestine tissue blocks were longitudinally cut into 5 µm sections by a rotary microtome (Microm HM340E, Thermo Scientific, MA, USA). For the histological examination, sections from six mice were stained with hematoxylin and eosin and observed using a Leica DM3000 microsystems (Leica, Milton Keynes, UK). Three regions were selected for each section, and the villi length, crypt depth and intestinal wall thickness were measured. Data analysis was conducted using ImageJ software (v1.8.0, NIH, MA, USA).

### Gut microbiota analysis

Microbial community genomic DNA was extracted from each 12 fecal samples using the FastDNA Spin Kit for Soil (MP Biomedicals, CA, USA) according to the manufacturer’s instructions. The DNA extract was checked on 1% agarose gel, and the DNA concentration and quality were determined with a NanoDrop 2000 UV-vis spectrophotometer (Thermo Scientific, MA, USA). The hypervariable region V3-V4 of the bacterial 16S rRNA gene was amplified with primer pairs 338F (5′-ACTCCTACGGGAGGCAGCAG-3′) and 806R (5′-GGACTACHVGGGTWTCTAAT-3′) by an ABI GeneAmp 9700 PCR thermocycler (ABI, CA, USA). All amplicons were sequenced using the paired-end method on an Illumina NovaSeq PE250 platform (Illumina, San Diego, USA) according to the standard protocols by Majorbio Bio-Pharm Technology Co., Ltd. (Shanghai, China). Raw reads were deposited into the NCBI Sequence Read Archive (SRA) database with the Bioproject ID PRJNA796167.

Raw 16S rRNA gene sequencing reads were demultiplexed, quality-filtered using fastp version 0.20.0 (10), and were merged using FLASH version 1.2.7 (11). Operational taxonomic units (OTUs) with a 97% similarity cutoff were clustered using UPARSE version 7.1 (12), and chimeric sequences were identified and removed. Taxonomy assignment of each OTU representative sequence was analyzed by RDP Classifier version 2.2 (13) against the Silva database using a confidence threshold of 0.7.

### Metagenomic sequencing analysis

The DNA extracted from 12 samples was fragmented to an average size of approximately 400 bp using Covaris M220 (Gene Company Limited, MA, USA) to prepare paired-end libraries. The paired-end library was constructed using NEXTflex Rapid DNA-Seq (Bioo Scientific, TX, USA). Adapters containing the full complement of sequencing primer hybridization sites were ligated to the blunt end of fragments. Paired-end sequencing was performed on Illumina NovaSeq (Illumina Inc., CA, USA) at Majorbio Bio-Pharm Technology Co., Ltd. (Shanghai, China) using NovaSeq Reagent Kits X according to the manufacturer’s instructions (www.illumina.com). Sequence data associated with this project have been deposited in the NCBI SRA with the Bioproject ID PRJNA742232.

The Kyoto Encyclopedia of Genes and Genomes (KEGG) annotation was conducted using Diamond (14) (http://www.diamondsearch.org/index.php, version 0.8.35) against the KEGG database (http://www.genome.jp/keeg/, version 94.2) with an e-value cutoff of 1e − 5. Subsequently, different microbial genes were mapped to the CAZymes database (http://www.cazy.org/) and KEGG pathway to assess changes in functional metabolism. Linear discriminate analysis effect size (LEfSe) was used to identify bacterial species and functional capacities of the gut microbiome exhibiting significantly different abundances between the NC group and CS group. Correlations between the *C. sporogenes* treatment-associated bacterial species and glucolipid metabolism-associated function capacities of the gut microbiome were evaluated in the 12 samples with metagenomic sequencing data using permutational analysis of variance based on 9,999 permutations using the vegan package in R (v.3.5.1) (15). The significance threshold was set at FDR < 0.05.

### Metabolome profiling of fecal samples

The fecal samples were extracted by methanol (300 µL of pure methanol was added to 50 µL of samples), followed by analysis of the extracted supernatant (150 µL) using a sophisticated analytical technique combining liquid chromatography (LC) with electrospray ionization (ESI) and tandem mass spectrometry (MS/MS) system (LC-ESI-MS/MS system). For ESI coupled with a triple quadrupole-linear ion trap (QTRAP) mass spectrometerin tandem mass spectrometry (ESI-QTRAP-MS/MS) analysis, a linear ion trap (LIT) and triple quadrupole (QQQ) scans were acquired on a QTRAP mass spectrometer, QTRAP LC-MS/MS System, equipped with an ESI Turbo Ion-Spray interface, operating in positive and negative ion mode and controlled by Analyst 1.6.3 software (Sciex). Instrument tuning and mass calibration were performed using 10 and 100 µmol/L polypropylene glycol solutions in QQQ and LIT modes, respectively. A specific set of multiple reaction monitoring (MRM) transitions was monitored for each period according to the metabolites eluted within this period.

### Metabolomic data analysis

Unsupervised principal component analysis was performed by the function prcomp within the statistical software R. Significantly regulated metabolites between groups were determined based on the value of variable importance in projection ≥1 and absolute log2 (fold change) ≥1. To prevent overfitting, a permutation test with 200 permutations was performed. The identified metabolites were annotated using the KEGG compound database (http://www.kegg.jp/kegg/compound/), and the annotated metabolites were subsequently linked to the KEGG pathway database (http://www.kegg.jp/kegg/pathway.html). Pathways containing significantly regulated metabolites were mapped and then were fed into metabolite sets enrichment analysis, with their significance assessed by the hypergeometric test’s *P*-values. The correlation coefficient was calculated using the Spearman’s rank correlation. The heatmap was plotted using the gplots package in R (v.3.5.1) (16).

### cDNA synthesis and real-time quantification PCR (RT-qPCR)

RNA extraction and reverse transcription were performed following the manufacturer’s instructions (Takara). Subsequently, the resulting cDNA was subjected to RT-qPCR using gene-specific primers (primer sequences were listed in Table S1) and TB Green Premix Ex Taq II in the presence of the StepOnePlus Real-Time PCR System (Applied Biosystems). Relative mRNA expression levels were determined using the 2^-ΔΔCT^ method, with glyceraldehyde-phosphate dehydrogenase (GAPDH) serving as the endogenous control in mouse samples.

### Protein extraction and western blot assay

Total protein was extracted using a radioimmunoprecipitation assay (RIPA) buffer (Beyotime, Shanghai, China) supplemented with phenylmethanesulfonyl fluoride (PMSF, Solarbio, Beijing, China). The supernatant was collected and quantified with a bovine serum albumin (BCA) protein assay kit (Cwbiotech, Beijing, China). Protein samples were separated by SDS-polyacrylamide gel electrophoresis (Thermo Scientific, MA, USA) and transferred onto polyvinylidene difluoride membranes (PVDF, Millipore, MA, USA). The PVDF membranes were then blocked with 5% skim milk in TBS-T buffer (a Tris-buffered saline with Tween-20) at room temperature for 1 h, followed by overnight incubation with primary antibodies at 4°C. Afterward, the membranes were probed with secondary antibodies at room temperature for 1 h. ACSS2 polyclonal antibody is used in the trial (16087-1-AP, Proteintech, Wuhan, China). Protein bands were visualized using a Gel Image System Bio-Rad (Hercules, CA, USA).

### Statistical analysis

Data are shown as means ± SEM. Statistical significance was determined using the two-tailed Student’s *t*-test. All analyses were performed using GraphPad PRISM v.8.02 (GraphPad Software). Results were considered statistically significant at *P < 0.05, **P < 0.01, and ***P < 0.001.

The linear discriminant analysis (LDA) effect size analysis (LDA score >2.5, *P*-value <0.05) was used to distinguish the differential fecal microbiota (DFMs) between the two groups at the class to genus level. The abundances of microbial metabolic pathways, modules, KEGG enzymes, and CAZymes were compared between the two groups using the Wilcoxon rank-sum test or LEfSe. Significant differences were determined by the FDR adjusted *P*-value <0.05, or an LDA score > 2 and *P*-value < 0.05.

## RESULTS

### *C. sporogenes* supplementation increased fat accumulation in mice by reducing the energy content excreted in feces

An intragastric experiment was performed to investigate the effect of *C. sporogenes* supplementation on body fat content in mice, as illustrated in Fig. 1A. Compared to mice in the NC group, those receiving *C. sporogenes* supplementation exhibited a 9.09% increase in body weight over 6 weeks (Fig. 1B). Notably, mice treated with *C. sporogenes* showed a significant increase in fat mass, with a 33.85% elevation (Fig. 1C). Specifically, the weight of eWAT was significantly increased by 36.69% (Fig. 1D and E, *P* < 0.01). Moreover, the adipocytes mean diameter and surface area in both eWAT and liver tissues of *C. sporogenes* treated mice were significantly larger than those in the control mice (Fig. 1F through H). Additionally, the sBAT exhibited a white fat phenotype following the *C. sporogenes* treatment (Fig. S1A). Circulating levels of TG and LDL-C in the CS group mice were significantly increased, while levels of TC and HDL-C remained unaffected (Fig. S1B).

**Fig 2:**
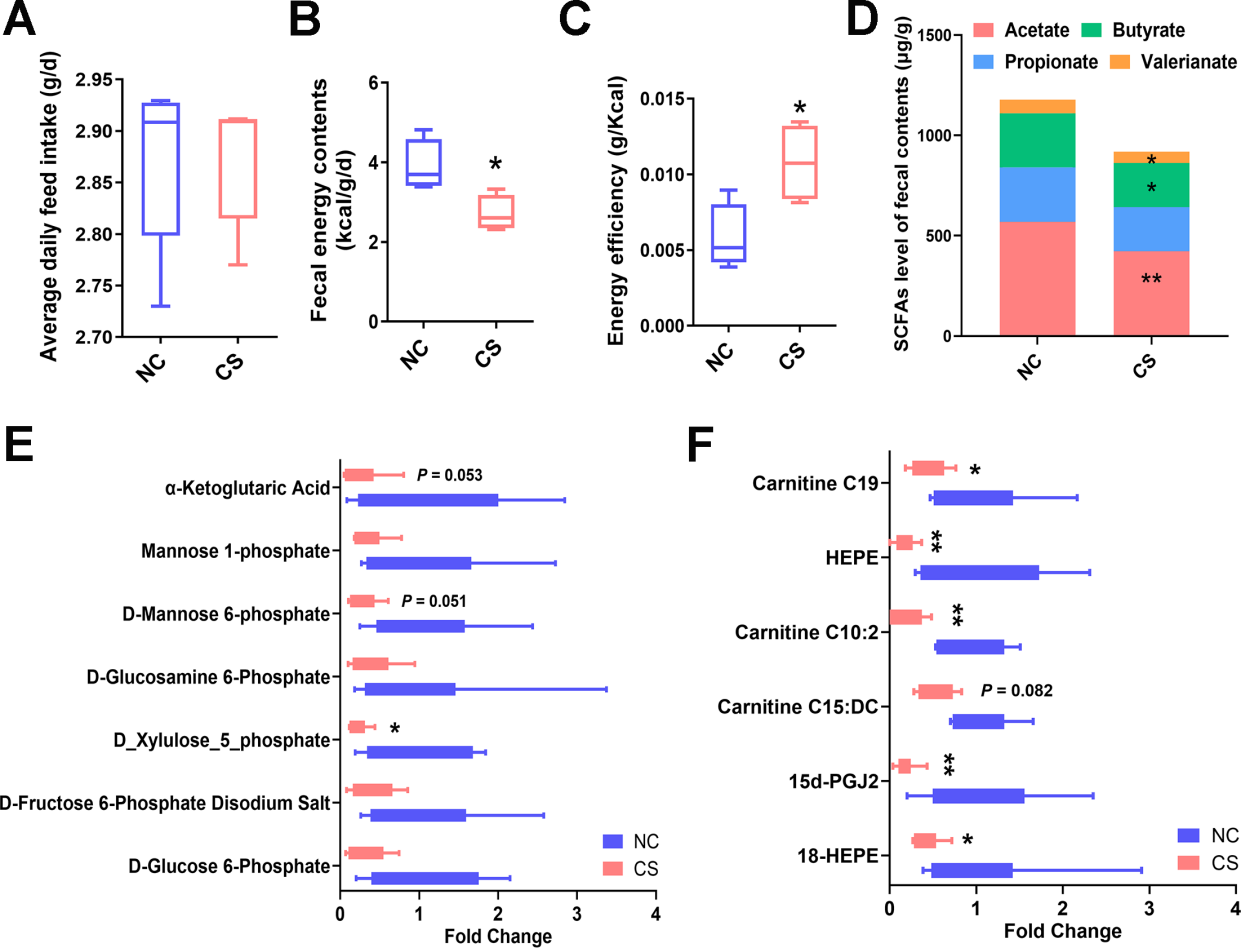
*C. sporogenes* treatment enhances energy absorption in mice. (**A**) Daily food intake per mouse. (**B**) Fecal energy contents and (**C**) host energy efficiency, calculated on the basis of the feces during the first 5 weeks of oral treatment (*n* = 10). (**D**) SCFA levels of feces in the mice after 42 days of treatment. (**E**) Fold change of the fecal carbohydrates and (**F**) fatty acid contents in mice of two groups after 42 days of treatment (*n* = 6). Data are presented as the mean ± SEM, *P < 0.05, **P < 0.01. (*n* = 6–10).

These findings suggest that *C. sporogenes* supplementation increased mice’s energy absorption and fat accumulation. The mice body weight gain was significantly elevated in the *C. sporogenes* treated group than those in the control group, but there were no significant differences in feed intake between the two groups (Fig. 2A). Interestingly, the fecal energy content of the treatment group exhibited a 30.44% decrease compared to the control group (Fig. 2B). Notably, compared to the control group, the energy efficiency of the *C. sporogenes* treated group increased significantly by 86.01% (Fig. 2C). To assess microbial energy consumption in the mouse intestine, concentrations of fecal SCFAs were detected. It was found that the acetate, propionate, butyrate, and valerate levels were reduced to varying degrees in the *C. sporogenes* treated mice, with acetate exhibiting the most significant decrease (Fig. 2D), indicating higher energy absorption in the treated mice.

To identify the underlying factors responsible for fat accumulation, dissimilarities of caloric content in fecal metabolites between the two groups of mice were determined by the metabolomics analysis. The orthogonal partial least squares discriminant analysis (OPLS-DA) score plot from the multivariate analysis revealed distinct clustering between the fecal samples of mice treated with and without *C. sporogenes*, indicating notable variation in metabolite composition between the two groups of mice (Fig. S2A). Specifically, out of 645 metabolites detected, 32 compounds showed significant alterations in abundances. Among these, four compounds were increased, while 28 compounds were significantly reduced in the mice treated with *C. sporogenes* (Fig. S2B). A clustering heatmap of the differentially expressed metabolites indicated that the primary classes involved were nucleotides, fatty acids, and amino acids with their respective metabolites (Fig. S2C).

Comparing metabolic profiles, it was found that the shift induced by *C. sporogenes* was mainly linked to carbohydrates and fatty acids, as indicated by OPLS-DA model coefficients. In comparison to control mice, CS group mice exhibited significantly decreased fecal carbohydrates such as D-mannose 6-phosphate (M6P), D-xylulose 5-phosphate (X5P), and α-ketoglutaric acid by 4.51-, 3.63-, and 3.58-folds, respectively (Fig. 2E). Several fatty acids, including hydroxyeicosapentaenoic acid (HEPE), (±)-18-hydroxy-5Z,8Z,11Z,14Z,16E-eicosapentaenoic acid (18-HEPE), and 15-deoxy-δ-12,14-PGJ2 (15d-PGJ2), showed marked reductions in the feces of CS group mice (Fig. 2F, *P* < 0.01).

### *C. sporogene*s supplementation resulted in improvements in intestinal structure and upregulation of glycolipid transporters in mice

**Fig 3:**
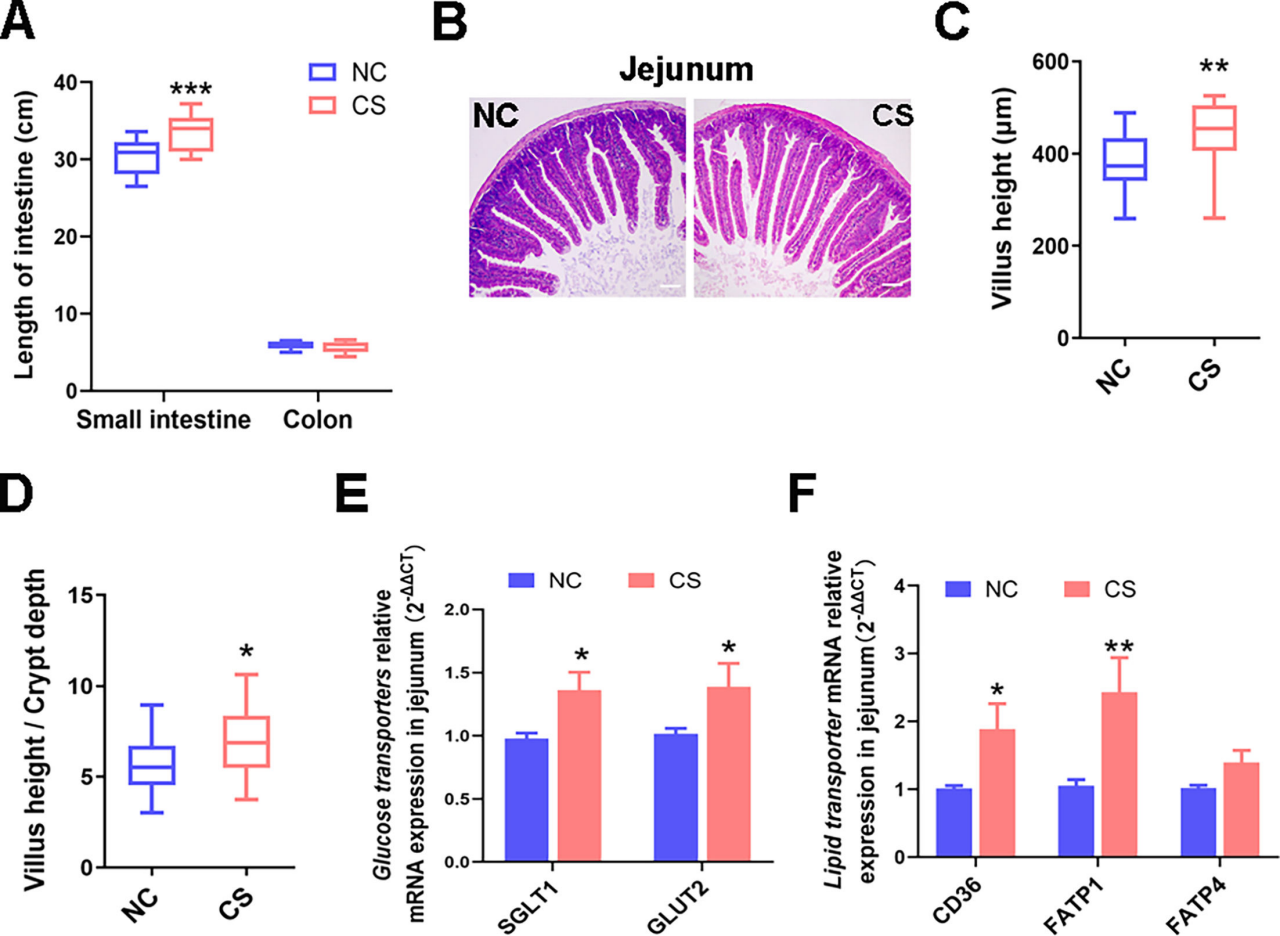
*C. sporogenes* treatment improves intestinal morphology and increased the gene expression levels of glycolipid transporters after 42 days of treatment. (**A**) Length of small intestine and colon in mice. (**B**) Representative hematoxylin-eosin-stained picture of jejunum tissue (×100). Scale bar, 100 µm. (**C**) Mean villus height and (**D**) the ratio of villus height and crypt depth of jejunal villi. (**E**) Relative mRNA expression levels of glucose transporters *SGLT1* and *GLUT2* in the jejunum by qRT-PCR. (**F**) Relative mRNA expression levels of lipid transports *FATP1*, *FATP4*, and *CD36* in the jejunum by qRT-PCR. Data are presented as the mean ± SEM, *P < 0.05, **P < 0.01, ***P < 0.001. (*n* = 6).

The enhanced energy absorption may be attributed to the augmented intestinal morphology and functionality. Our findings revealed a notable increase in the total length of the small intestine (including the duodenum, jejunum, and ileum) in mice of the CS group compared to those in the NC group (Fig. 3A). Histological analysis of the jejunum, a crucial site for nutrient absorption (17), showed a significant increase in jejunal villus height and the ratio of intestinal villi to crypt in mice from the CS group (Fig. 3B through D), indicating an expansion of the absorptive surface area in the foregut.

Furthermore, transcriptional levels of jejunal glycolipid transporters revealed a significant increase in the expression of glucose transporter genes, sodium-dependent glucose transporter 1 (*SGLT1*), and glucose transporter 2 (*GLUT2*) (Fig. 3E) as well as fatty acid transporter genes (*FAT/CD36*), fatty acid transport protein 1 (*FATP1*), and *FATP4* in the jejunum of CS group mice (Fig. 3F). These results confirm that *C. sporogenes* supplementation enhances intestinal morphology and improved the absorption capacity for intestinal nutrients and energy in mice.

### *C. sporogenes* supplementation promoted adipogenesis and hepatic glycolipid metabolism

**Fig 4:**
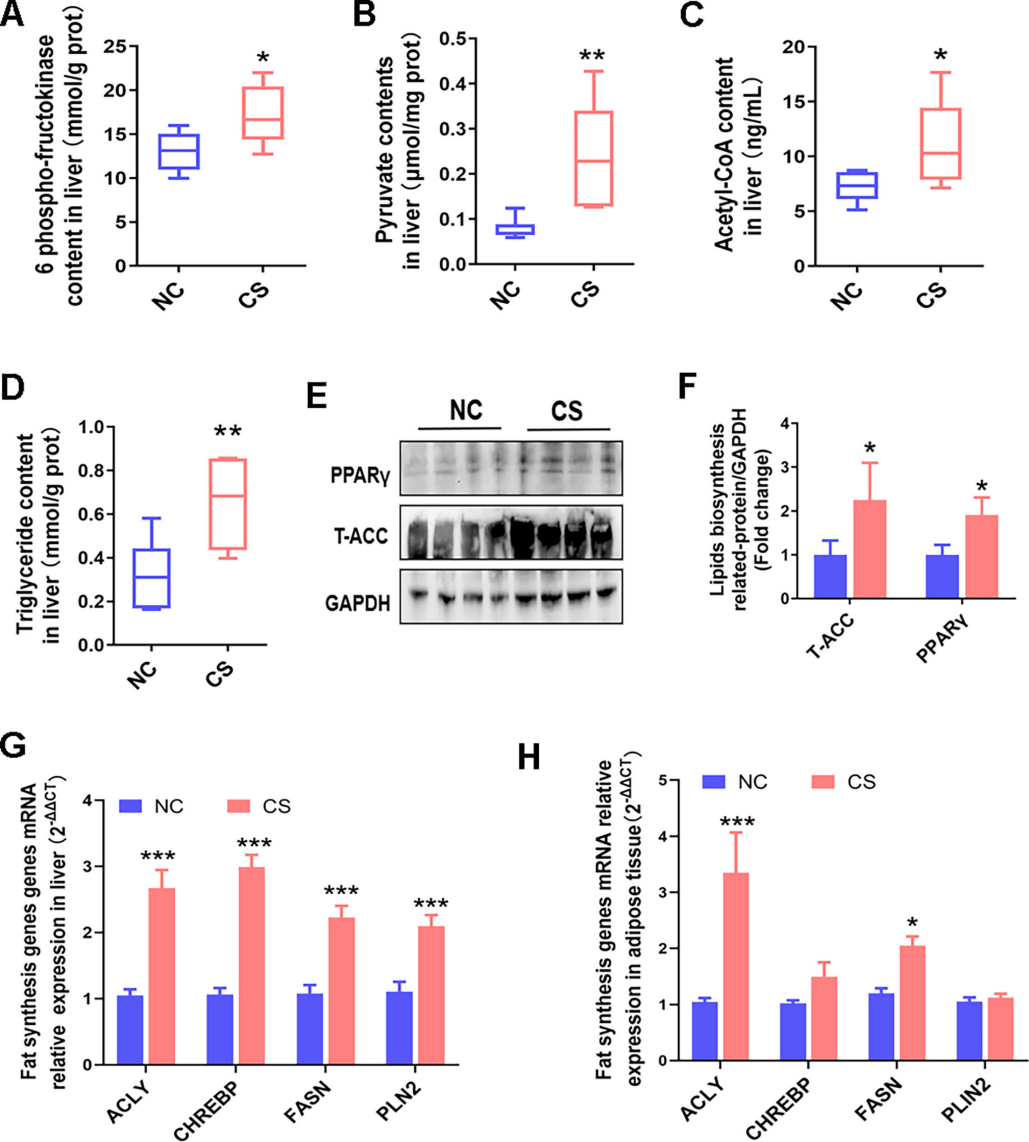
*C. sporogenes* promotes glycolipids biosynthesis in mice after 42 days of oral treatment. (**A**) Levels of key rate-limiting enzyme, PFK, and (**B**) the end product pyruvate in glycolysis. (**C**) Contents of precursor substance for lipogenesis, hepatic acetyl-CoA, and (**D**) TG. (**E**) Relative hepatic protein expression levels of T-ACC and (**F**) PPARγ, which are required for lipogenesis in liver. (**G**) Relative mRNA expression levels of fat synthesis genes *ACLY*, *CHREBP*, *FASN*, and *PLIN2* by qRT-PCR after 42 days of treatment in the liver and (**H**) eWAT. Data are presented as the mean ± SEM, *P < 0.05, **P < 0.01, ***P < 0.001. (*n* = 6).

Assessment of key glycolysis-related enzymes and metabolites in the liver showed that glycolysis-related products, including pyruvate, were significantly elevated in the CS group mice compared to the NC group mice (Fig. 4A and B). Additionally, concentrations of acetyl-CoA, a precursor of fat synthesis, and TG in the liver of CS group mice were substantially higher than those in NC group mice (Fig. 4C and D), indicating that *C. sporogenes* supplementation increases the precursors for fat synthesis in the liver.

The hepatic total acetyl-CoA carboxylase (T-ACC), a rate-limiting enzyme in *de novo* fatty acid synthesis (18), was found to have increased 2.25-fold in mice with the *C. sporogenes* treatment. Additionally, the *C. sporogenes* treatment led to a significant increase in the level of peroxisome proliferator-activated receptor-gamma (PPARγ) protein, which is an essential regulator for adipocyte differentiation and lipid storage (19) (Fig. 4E and F).

Analysis of the gene expression levels of regulators involved in lipogenesis in the liver and adipose tissues further revealed upregulated expression of lipogenesis-related genes, including ATP citrate lyase (*ACLY*), carbohydrate-responsive element binding protein (*CHREBP*), fatty acid synthase (*FASN*), and perilipin 2 (*PLIN2*) in the liver of *C. sporogenes* treated mice compared to the control mice (Fig. 4G). Similarly, the expression levels of *ACLY* and *FASN* genes in eWAT were significantly increased in the CS group mice (Fig. 4H). These results suggest that *C. sporogenes* effectively promotes adipogenesis in mice.

### *C. sporogenes* supplementation elevated the abundance of *Clostridium* bacteria in the intestines

**Fig 5:**
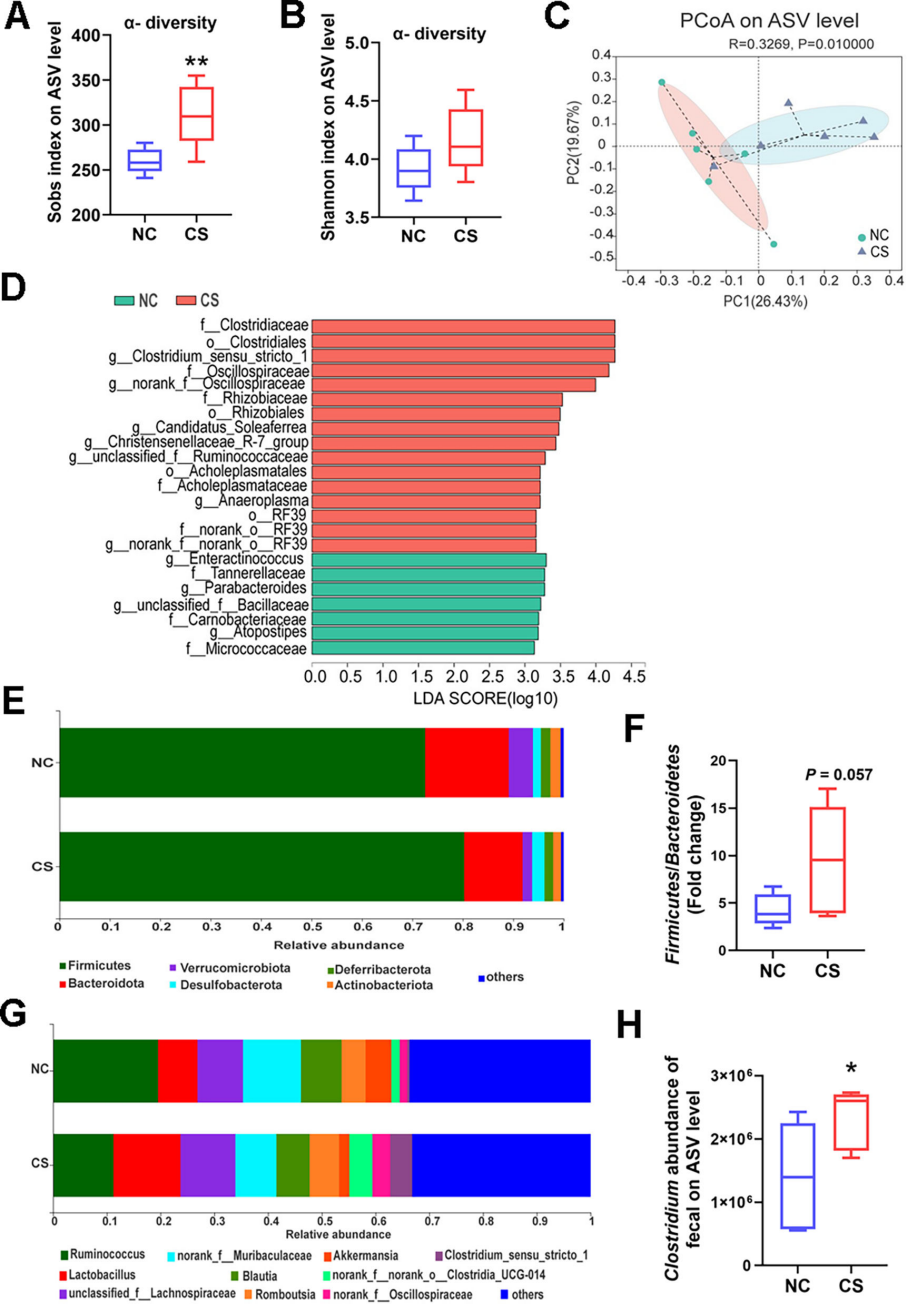
*C. sporogenes* leads to higher microbial diversity and *Clostridium* genus abundances in feces by 16S rRNA sequencing. (**A**) α-Diversity index comparisons of Sob index and (**B**) Shannon index. (**C**) Principal coordinate analysis (PCoA) analysis of microbes from mice of both groups. (**D**) LEfSe results of the main microbes. (**E**) Microbial composition at the phylum level and (**F**) the ratio of Firmicutes and Bacteroidetes. (**G**) Microbial composition at the genus level and (**H**) the abundance of *Clostridium* genus. Data are presented as the mean ± SEM, *P < 0.05, **P < 0.01. (*n* = 6).

To investigate the association between *C. sporogenes* and body energy metabolism, we analyzed fecal microbial composition and energy homeostasis in mice. The α-diversity of fecal microbiota in the CS group of mice showed a significant increase, particularly in the Sob index, compared to the control group mice (Fig. 5A). However, no significant difference was observed in the Shannon index between groups (Fig. 5B). The β-diversity analysis revealed a distinct difference in the gut microbial composition of *C. sporogenes* treated mice compared to controls (Fig. 5C). Additionally, a total of 421 amplicon sequence variants (ASVs) were identified through sequencing. Notably, *C. sporogenes* treated mice exhibited greater diversity and richness in gut bacterial community compared to control mice, with the control group mice having 145 unique ASVs, while the C. *sporogenes* treated mice had 224 unique ASVs (Fig. S3A).

The LEfSe analysis was used to estimate specific microbial signatures between the two groups. Twenty-three discriminative features were found, indicating higher levels of Clostridiales and Rhizobiales order, Clostridiaceae and Oscillatoriaceae family, and *Clostridium sensu stricto* 1 genus (Fig. 5D) in mice from the CS group. The *C. sporogenes* treatment led to an increase in the Firmicutes phylum and a decrease in the Bacteroidetes phylum, resulting in an overall increase in the F/B, a biomarker of host energy metabolism (Fig. 5E and F). Notably, the fecal *Clostridium* clusters (belonging to Firmicutes phylum) at the genus level were 1.66 times higher in *C. sporogenes* treated mice compared to the NC group (Fig. 5G and H).

**Fig 6:**
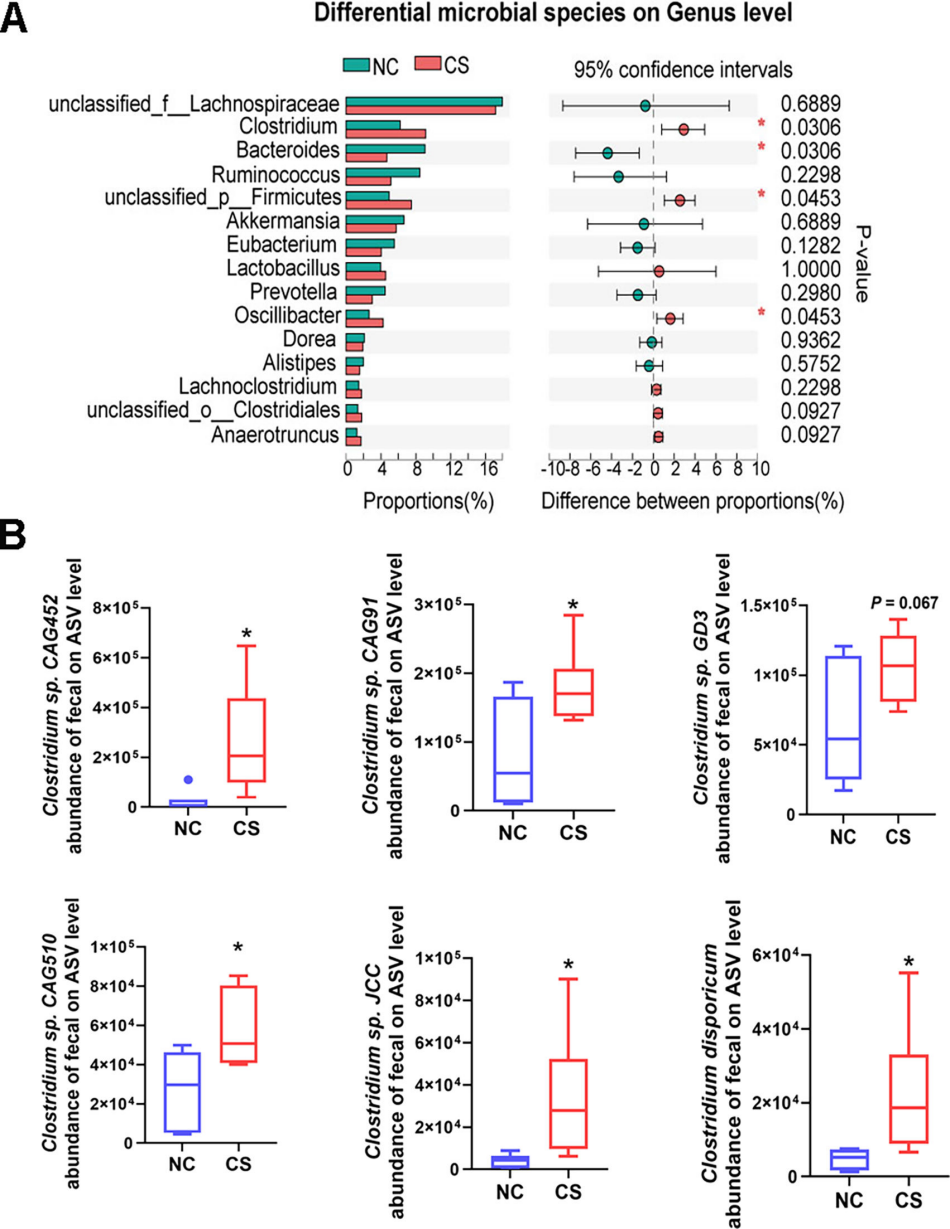
*C. sporogenes* significantly increases the abundance of *Clostridium* bacteria in feces by metagenomic sequencing. (**A**) Differential fecal microbes in feces of two groups, and statistical significance of difference was determined using Wilcoxon rank-sum test. (**B**) Comparison of *Clostridium* bacteria at species level, data are presented as the mean ± SEM, *P < 0.05. (*n* = 6).

The variability in gut microbiota between the two groups was further assessed through metagenomic profiling of DFMs composition and function, yielding results mostly consistent with 16S rDNA sequencing. At the genus level, mice treated with *C. sporogenes* showed a significant increase in *Clostridium* and *Oscillibacter*, along with a significant decrease in *Bacteroides* (Fig. 6A). At the species level, differential strains between groups predominantly belonged to the *Clostridium* genus (Fig. S3B). Quantitative analysis based on ASVs level revealed a significant increase in *Clostridium* sp. CAG:452, *Clostridium* sp. JCC, and *Clostridium disporicum* with increases of 10.79-fold, 8.47-fold, and 4.75-fold, respectively, compared to control mice (refer to Fig. 6B for details). Additionally, the quantitative results showed that the abundance of *C. sporogenes* in the feces of treated mice increased by 37.15%, but the result was not significant (Fig. S3C). All the results demonstrate that *C. sporogene*s supplementation significantly augments the abundance of *Clostridium* microbes associated with regulating host energy metabolism in the gut of mice.

### The increase of *Clostridium* bacteria is a contributing factor to changes in glycolipid metabolism and energy homeostasis

**Fig 7:**
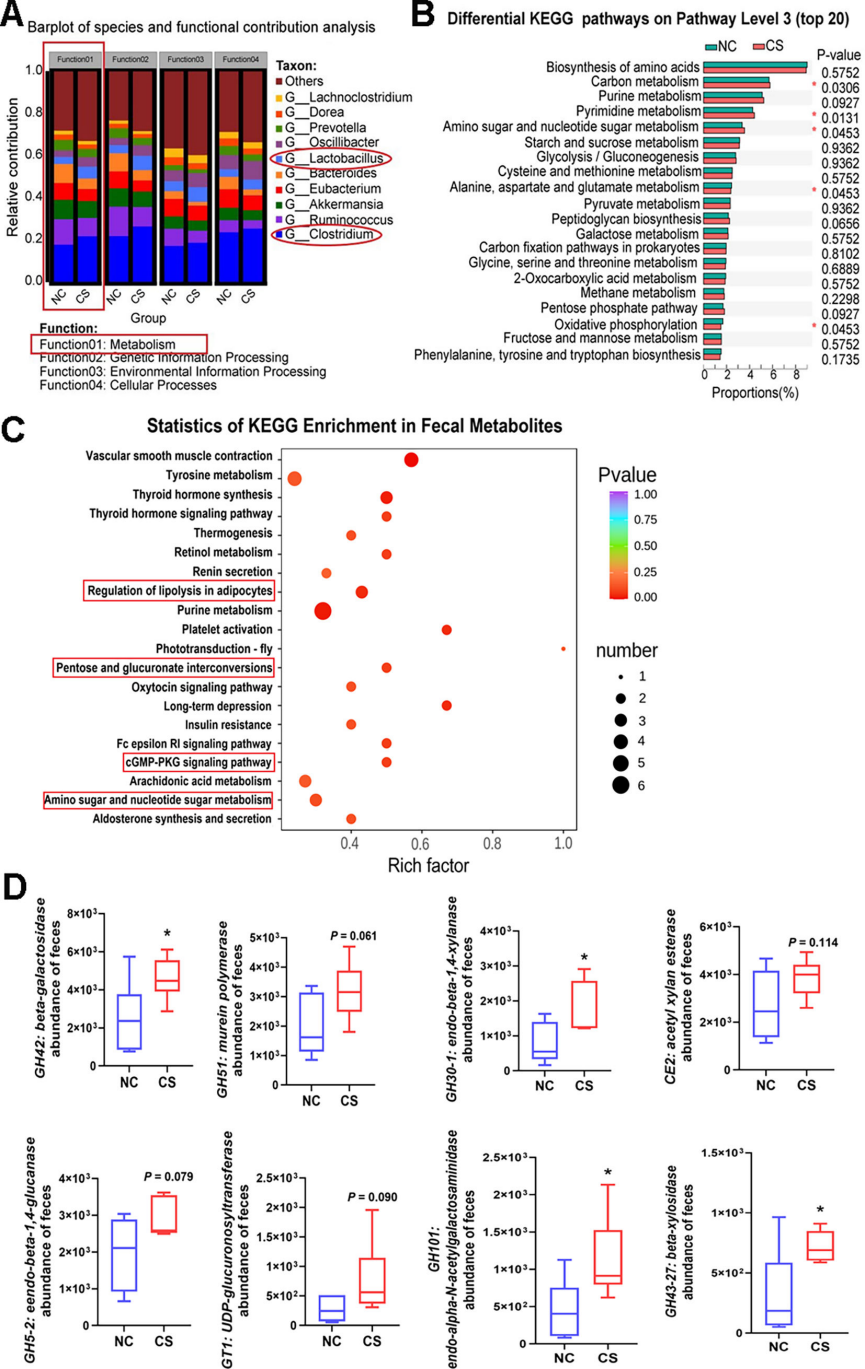
Effect of increased *Clostridium* bacteria on energy metabolism and CAZymes activity. (**A**) Relative contribution of different microbial genera to identified stool-enriched functional attributes. (**B**) Differential KEGG pathways at level 3 for the fecal microbiome, statistical significance of difference was determined using Wilcoxon rank-sum test. (**C**) Bubble diagram of KEGG enrichment analysis, multiple KEGG pathways related to glycolipid metabolism, and energy turnover were enhanced. (**D**) Relative abundance of predicted CAZymes in gut microbiota of the two groups, data are presented as the mean ± SEM, *P < 0.05. (*n* = 6).

To evaluate the impact of increased *Clostridium* cluster on metabolism, we analyzed microbial species’ contributions to the KEGG metabolism pathway using metagenomic data. The analysis revealed elevated levels of *Clostridium* and *Lactobacillus* genera in mice treated with *C. sporogenes*, indicating their significant influence on mouse metabolism (Fig. 7A). Five KEGG metabolic pathways differed between the two groups of mice. Specifically, carbon metabolism, pyrimidine metabolism, and amino sugar and nucleotide sugar metabolism showed increased activity, whereas alanine, aspartic acid, and glutamate metabolism along with oxidative phosphorylation demonstrated decreased activity in mice due to *C. sporogenes* treatment (Fig. 7B). Several KEGG pathways related to glycolipid metabolism and energy turnover were found to be enhanced, including pentose and glucuronate interconversions, amino sugar and nucleotide sugar metabolism, regulating lipolysis in adipocytes, and the cyclic guanosine monophosphate - protein kinase G (cGMP-PKG) signaling pathway (Fig. 7C). These findings confirm enhanced glycolipid metabolism and reduced energy expenditure in mice, possibly attributed to the significant increase in *Clostridium* species.

Meanwhile, differential analysis of all CAZymes categories was performed to evaluate the effects of *C. sporogenes* supplementation on carbohydrate metabolism. Twenty-one differential CAZymes were identified between the two groups, with 15 CAZymes significantly enriched in *C. sporogenes* treated mice, primarily involved in galactose, xylan, and glucan metabolism (Fig. S4A). Statistical analysis of CAZymes showed significant alternations in GH101, GH30-1, GH43-27, and GH43 in the feces of CS group mice, with values 2.45, 2.25, 2.25, and 1.81 times higher than those of the control group, respectively (Fig. 7D). Notably, phylogenetic analysis of CAZymes contigs showed that the genera *Clostridium*, *unclassified_f_Lachnospiraceae*, and *Bacteroides* mainly contributed to the CAZyme-encoding gene fragments of GH (glycoside hydrolases), carbohydrate esterases, glycosyltransferases, and carbohydrate-binding module families in the mouse fecal metagenome (Fig. S4B). These results also suggest that the increase in *Clostridium* bacteria resulting from *C. sporogenes* supplementation enhances glycolipid and carbohydrate metabolism.

## DISCUSSION

Numerous studies have suggested that *Clostridium* bacteria may contribute to host fat accumulation, yet the specific microbial species and underlying mechanisms remain incompletely understood. *C. sporogenes*, an anaerobic strain of the *Clostridium* genus in the Firmicutes phylum, is known to produce energy-rich metabolites through the fermentation of amino acids and other nutrients (9). In our study, *C. sporogenes* supplementation increased body fat and enhanced lipogenesis in both adipose and liver tissues of mice. Given that there was no significant change in feed intake, it is evident that fat deposition in mice was a consequence of enhanced nutrient absorption in the gut (20). KEGG enrichment analysis of differential microbiota and metabolites revealed that supplementing with *C. sporogenes* significantly promotes pathways regulating energy metabolism, establishing a positive correlation between the *Clostridium* genus and carbohydrate metabolism (21). Thus, our study suggested that *C. sporogenes* promotes adipogenesis and fat accumulation in mice by enhancing the metabolic absorption of carbohydrates.

Furthermore, we observed that *C. sporogenes* altered the microbial composition of mice, primarily by increasing the α-diversity of gut microbiota, particularly the F/B ratio. The ratio of Firmicutes to Bacteroidetes serves as a marker for metabolizing carbohydrates into acetate compounds (22, 23), which is also positively associated with host growth and development (24, 25). Most studies suggest that individuals with a higher F/B ratio exhibit greater efficiency of gut microbiota in extracting energy from the diet (2). Firmicutes utilize free enzymes and cellulosomes for the conversion of plant polysaccharides into SCFAs, particularly *Clostridium* cluster IV and *Clostridium* cluster XIVa within the Firmicutes phylum, which are abundant and prevalent in the intestinal microbiota of adult animals and serve as main producers of the energy substance butyrate (26, 27). β-Galactosidase (GH2 and GH42) ferment lactose to promote energy harvesting in Firmicutes (28), consistent with a 1.81-fold increase in GH42 observed in the gut microbiota of mice in the treated group in our study. Therefore, *C. sporogenes* may facilitate fat storage in mice by increasing the abundance of *Clostridium* microbes.

Significant differences were observed in fecal carbohydrates and their derivatives between the two groups in this study. Specifically, fecal excretions of M6P and X5P were significantly diminished in the CS group mice, suggesting a potential promotion of carbohydrate absorption by *Clostridium*. Intestinal M6P and X5P undergo conversion into fructose-6-phosphate via the hexose monophosphate pathway. Subsequently, pyruvate and acetyl-CoA are produced through the glycolysis pathway, both of which play vital roles in lipid synthesis (29). Furthermore, the concentrations of fecal fatty acids (HEPE, 18-HEPE, and 15d-PGJ2) were significantly reduced in mice treated with *C. sporogenes*, indicating their potential utilization in hepatic lipid synthesis. Previous research have indicated that various PPARs are activated by HEPE, 18-HEPE, and 15d-PGJ2 (30). Specifically, 15d-PGJ2 can activate the adipocyte determination factor PPARγ, ultimately leading to increased fat accumulation (31, 32). Thus, there is compelling evidence to suggest that *Clostridium* species may increase the absorption of carbohydrates M6P and X5P, as well as promote the uptake of fatty acids HEPE, 18-HEPE, and 15d-PGJ2 for fat storage in adipose tissue, thereby contributing to their growth-promoting effect.

Consequently, *C. sporogenes* supplementation may be associated with promoting growth and fat deposition in mice by augmenting the abundance of *Clostridium* species. Our findings lay the groundwork for future investigations into the interplay between *Clostridium* bacteria, energy metabolism, and obesity. Moreover, the data underscore the potential benefits of targeting gut microbiota through the strategic use of specific bacteria to modulate host energy metabolism.

### Conclusion

In summary, *C. sporogenes* supplementation notably augmented the abundance of *Clostridium* bacteria, thereby enhancing the metabolic uptake of carbohydrates and fatty acids, ultimately leading to adipogenesis and the accumulation of body fat in mice.

## SUPPLEMENTAL MATERIAL

Fig. S1 spectrum.04116-23-s0001.tif Lipids deposition in liver, subscapular brown adipose (sBAT) and serum.

Fig. S2 spectrum.04116-23-s0002.tif Fecal microbiome composition after 42 days of treatment.

Fig. S3 spectrum.04116-23-s0003.tif Fecal microbiome composition in mice after 42 days of oral treatment.

Fig. S4 spectrum.04116-23-s0004.tif KEGG functional analysis of microbiome.

Supplemental material spectrum.04116-23-s0005.pdf Legends for Fig. S1 to S4.

Table S1 spectrum.04116-23-s0006.doc Gene primer sequences.

ASM does not own the copyrights to Supplemental Material that may be linked to, or accessed through, an article. The authors have granted ASM a non-exclusive, world-wide license to publish the Supplemental Material files. Please contact the corresponding author directly for reuse.
