# Supplementary figures and images for: Clostridium sporogenes increases fat accumulation in mice by enhancing energy absorption and adipogenesis
Source: Microbiol Spectr. 2024 Jun 25;12(8):e04116-23. doi: 10.1128/spectrum.04116-23 (PMC11302664; doi:10.1128/spectrum.04116-23)

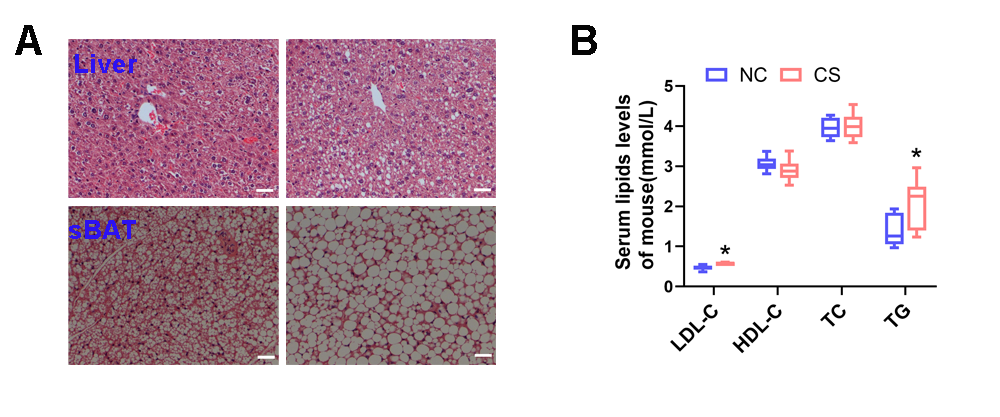

Supplement: Fig. S1 — Lipids deposition in liver, subscapular brown adipose (sBAT) and serum. [file spectrum.04116-23-s0001.tif]

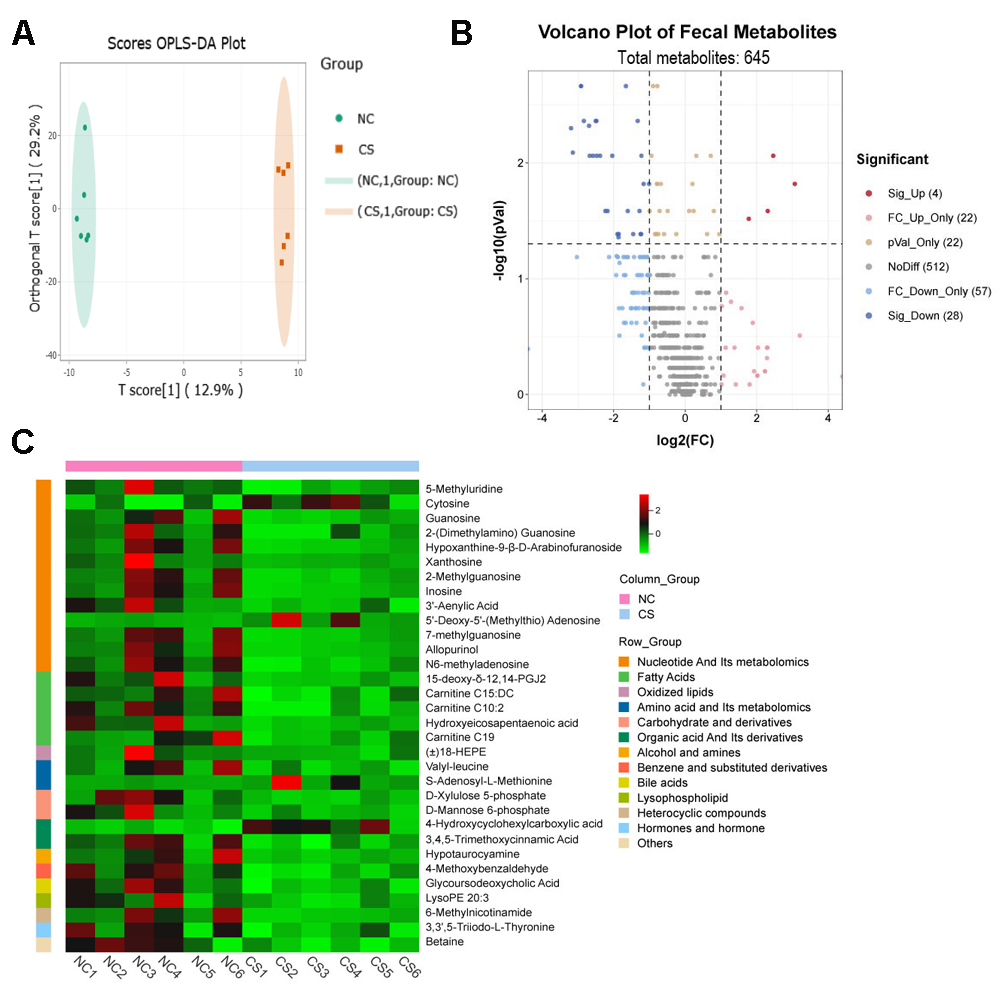

Supplement: Fig. S2 — Fecal microbiome composition after 42 days of treatment. [file spectrum.04116-23-s0002.tif]

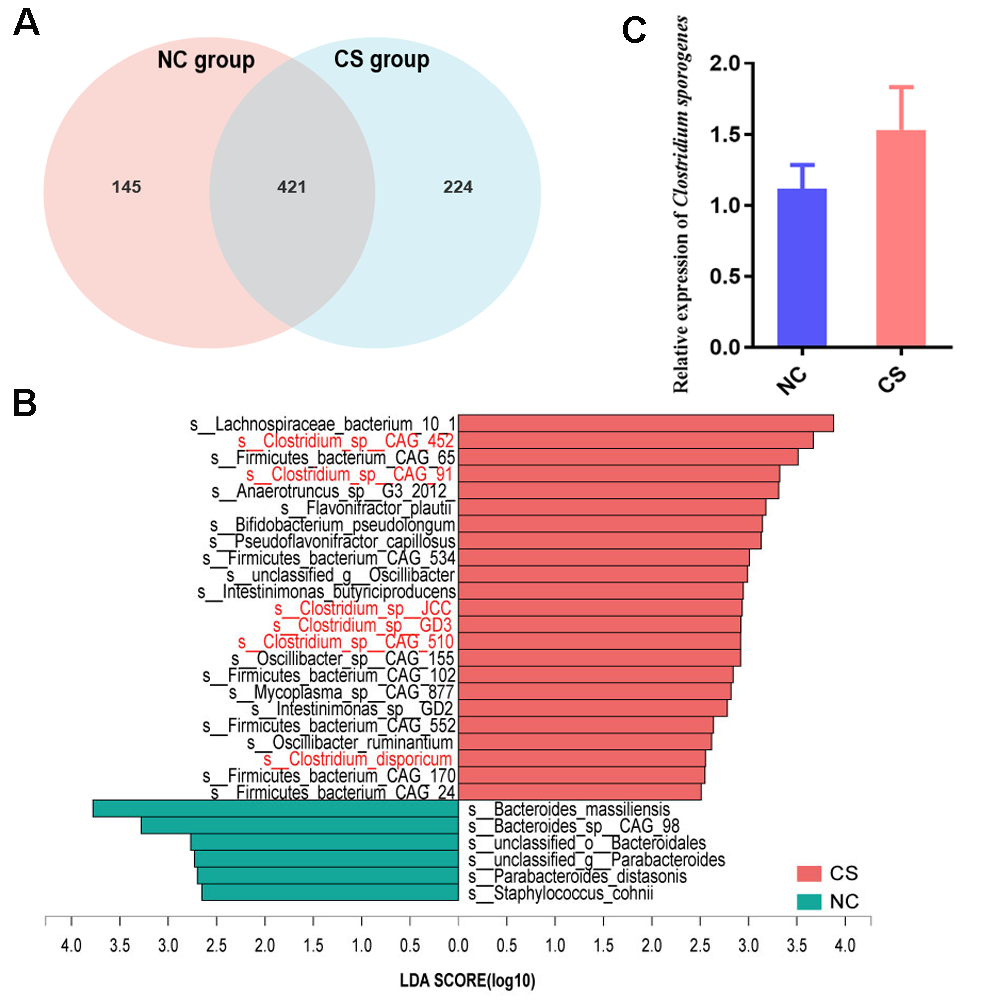

Supplement: Fig. S3 — Fecal microbiome composition in mice after 42 days of oral treatment. [file spectrum.04116-23-s0003.tif]

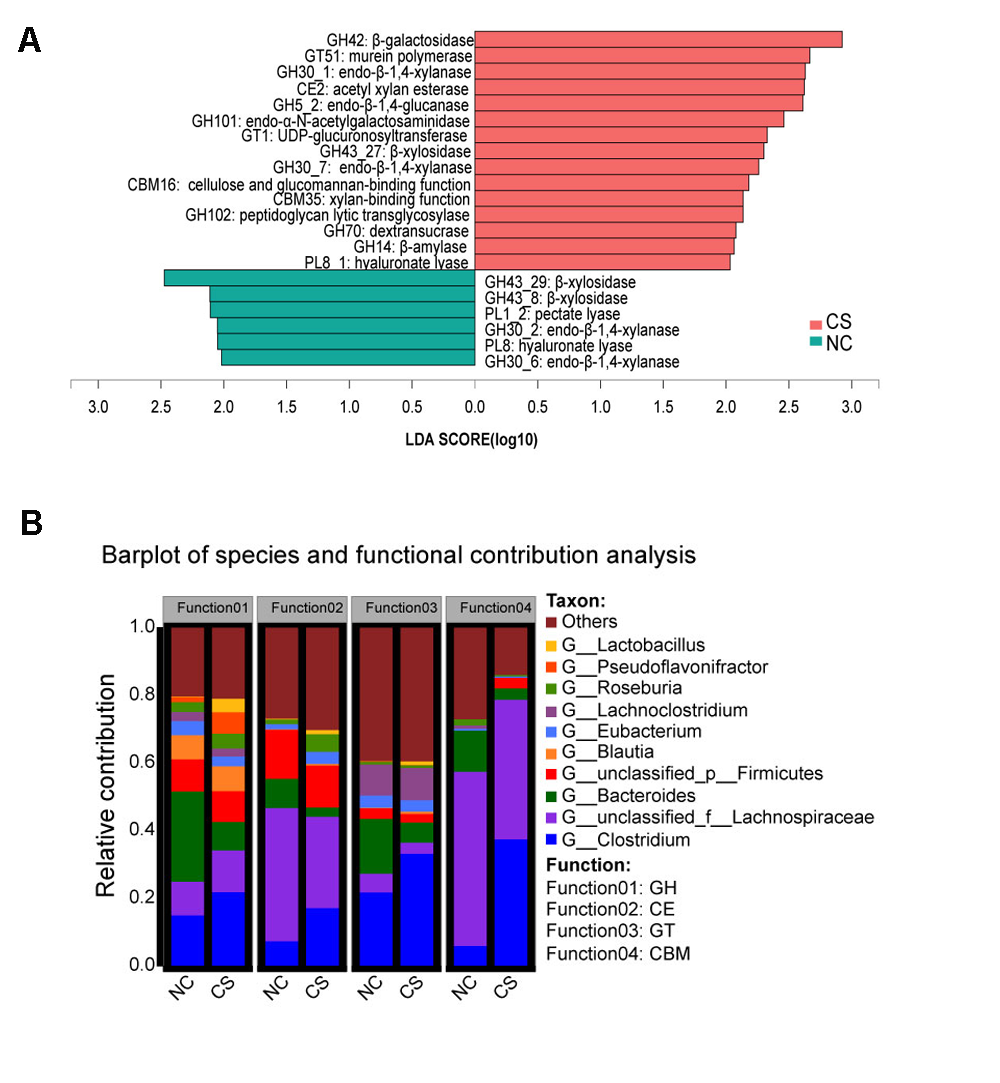

Supplement: Fig. S4 — KEGG functional analysis of microbiome. [file spectrum.04116-23-s0004.tif]
