# Supplementary material for: Clostridium sporogenes increases fat accumulation in mice by enhancing energy absorption and adipogenesis
Source: Microbiol Spectr. 2024 Jun 25;12(8):e04116-23. doi: 10.1128/spectrum.04116-23 (PMC11302664; doi:10.1128/spectrum.04116-23)
Supplement: Supplemental material — Legends for Fig. S1 to S4. [file spectrum.04116-23-s0005.pdf]

Figure legend

Figure S1 Lipids accumulation levels of liver, subscapular brown adipose (sBAT) tissues and serum in mice after 42 days of oral treatment. (A) Representative HE-stained picture of liver and sBAT tissues (× 100), Scale bar, 50 μm. (B) Contents of serum lipids, low-density lipoprotein cholesterol (LDL-C), HDL-C, total cholesterol (TC) and triglyceride (TG). Data are presented as the mean ± SEM, *P < 0.05. (n = 6)

Figure S2 Fecal microbiome composition in mice after 42 days of oral treatment. (A) Total ASVs from the sequencing in all mice were displayed by Venn diagram. (B) Differential strains between the groups at the species level, most of them belonged to the *Clostridium* genus. (n = 6)

Figure S3 Fecal microbiome composition in mice after 42 days of oral treatment. (A) Total ASVs from the sequencing in all mice were displayed by Venn diagram. (B) Differential strains between the groups at the species level, most of them belonged to the *Clostridium* genus. (C) The relative expression level of *C. sporogenes* in feces of mice were analyzed by qRT-PCR. (n = 6)

Figure S4 KEGG functional analysis of microbiome. (A) Relative contribution of different CAZymes categories to the changes of *C. sporogenes* supplementation on carbohydrate metabolism. (B) Relative contribution of different microbial genera to predicted CAZymes. (n = 6)
