# Supplementary material for: Clostridium sporogenes increases fat accumulation in mice by enhancing energy absorption and adipogenesis
Source: Microbiol Spectr. 2024 Jun 25;12(8):e04116-23. doi: 10.1128/spectrum.04116-23 (PMC11302664; doi:10.1128/spectrum.04116-23)
Supplement: Table S1 — Gene primer sequences. [file spectrum.04116-23-s0006.doc]

| **Table S1 Genes Primer Sequences** | |  |
| --- | --- | --- |
| Gene name | Primer | Sequence (5'→3') |
| GAPDH | F | GATGGGCGTGAACCATGAGA |
|  | R | GATGCCGAAGTGGTCATGGA |
| ACLY | F | GCTGCCATGGTCTACCCTTT |
|  | R | GATCAGCACGTCTACCTCCG |
| CHREBP | F | TTCCTGCACCCACAACCTTT |
|  | R | TTGCATACCCTGAGGCACAG |
| FASN | F | CAAGTGTCCACCAACAAGCG |
|  | R | GGAGCGCAGGATAGACTCAC |
| PLIN2 | F | TGGTGATGAGAGTGGCCAAC |
|  | R | TTCTCGGCCATCTCACACAC |
| CD36 | F | TAGTAGAACCGGGCCACGTA |
|  | R | CAGCCAGGACTGCACCAATA |
| FATP4 | F | TCAATAGCCGCATCCTGTCC |
|  | R | AGAGGGTCCTGCTGGATGAT |
| FATP1 | F | CGCCGATGTGCTCTATGACT |
|  | R | ACACAGTCATCCCAGAAGCG |
| SGLT1 | F | CACCGAGGGCTGACTCATTC |
|  | R | TGATCCGTACACCAGTACCAC |
| GLUT2 | F | TGCTGCTGGATAAATTCGCC |
|  | R | GCAGAGGGCGATGACAAAAT |
